# Supplementary material for: Advancing interactive systems with liquid crystal network-based adaptive electronics
Source: Nat Commun. 2024 May 17;15:4191. doi: 10.1038/s41467-024-48353-7 (PMC11101476; doi:10.1038/s41467-024-48353-7)
Supplement: Supplementary file 1 — Supplementary Information [file 41467_2024_48353_MOESM1_ESM.pdf]

Supplementary Information

Advancing Interactive Systems with Liquid Crystal Network-Based Adaptive Electronics

Pengrong Lyu[1,2], Dirk J Broer[1,2], Danqing Liu [1,2]✉

[1] Institute for Complex Molecular Systems, Eindhoven University of Technology, Den Dolech 2, 5612 AZ, Eindhoven, The Netherlands.

[2] Department of Chemical Engineering and Chemistry, Eindhoven University of Technology, Den Dolech 2, 5612 AZ, Eindhoven, The Netherlands.

✉Email: danqing.liu@tue.nl

Supplementary Note S1: Fabrication procedures for the adaptive electronic unit

(1) Preparation of the substrate with PVA

To prepare the PVA functionalized glass substrate, 76 × 52 × 1 mm precleaned microscope slides (Marienfeld, Germany) were used. 10 wt % PVA with a molecular weight of 9000 was dissolved in distilled water and spin-coated on the slides using a spin coater (Model WS-650MZ-23NPPB) rotating at 2000 rpm for 30 s. The PVA-coated glass plates were then placed on a hot plate (90 °C) for 10 min to evaporate the water.

(2) Printing the actuating electrode

A commercial 3D printer (EHR, Hyrel 3D) was used to print the heating electrode. Firstly, the conductive ink (a mixture containing 50 wt% PE874 and 50 wt% PE671, typically) was filled into the syringe and then loaded into the printer. The print head and bed temperature were set at ambient temperature (about 21 ^{o}C). A nozzle with a diameter of 150 μm was selected. The conductive ink was extruded into the PVA functionalized glass substrate at 200 mm/min speed following the pre-designed paths (Supplementary Fig. 1).

(3) Printing the sensing and auxiliary electrodes

After printing the actuating electrode, a new ink (pure Elepaste NP1) was filled into the syringe and then loaded into the printer. This ink was extruded onto the same glass substrate at 200 mm/min speed following the pre-designed path (Supplementary Fig. 1), using the same original position as for the printing process for the actuating electrode.

(4) Thermal curing of the conductive inks

After printing the two conductive inks on the substrate, they were placed in an oven (120 ^{o}C) for 20 min to fully cure the conductive inks.

(5) Printing the liquid crystal oligomer in the logic switch region

The liquid crystal oligomer (containing 60 m% Molecular **1**, typically) was filled into the syringe, which was loaded into the printer sequentially. The print head and bed temperature were set at 50 ^{o}C and 25 ^{o}C respectively. A nozzle with a diameter of 335 μm was selected. Then, the oligomer was extruded onto the substrate and over the printed electrodes at 400 mm/min speed following the pre-designed path. To achieve this, the same co-ordinate origin was used to initialize printing as was used for printing the actuation electrodes. Finally, the aligned oligomer was photo-crosslinked in a nitrogen box with UV light using an Omniture S2000 UV lamp (300 − 500 nm) at an intensity of 30 mW/cm^{-2} for 20 min. The temperature of the nitrogen box was set above room temperature depending on whether the sample being prepared was going to be used for investigating the influence of threshold temperature on activation, or if the sample was being prepared for construction of the artificial *Mimosa*.

(6) Printing the liquid crystal oligomer in the rest region

The same oligomer ink was printed in the rest region of the adaptive electronic unit using the same coordinate origin as the printed electrodes. The printing parameters (speed, print heat, and bed temperature) were kept the same as in Step (5). Photopolymerization was performed at ambient temperature (about 21 ^{o}C).

(7) Obtaining the free-standing printed film by dissolving PVA

After printing the electrodes and liquid crystal elastomer, the samples were immersed in deionized water for 3 hours to dissolve the PVA layer. The entire printed films were then

carefully peeled off from the substrate under the water. After further drying the sample at room temperature, a single adaptive electronics unit was obtained.

(8) Creating the micro-gap in the printed film
A homemade die was used to cut through both the sensing electrode and LCON film at a desired temperature (above room temperature). The homemade die was fabricated by cutting the blade (double edge razor blades, King C Gillette) into thin slices with a width of about 1 mm. Its thickness (in the edge) is less than 1 micron.

Supplementary Note S2: Finite Element Method (FEM) analysis of the adaptive logic switch

COMSOL Multiphysics (6.1 version) was used to simulate the thermal deformation of LCON and the resistance of the sensing electrode with varied temperatures. To do this, we need to couple two physics modules. Firstly, the Solid Mechanics module is used to analyze the thermal deformation behavior in LCON and the sensing electrode under different temperatures. Secondly, the Electric Currents module is used to calculate the electric potential distribution in the sensing electrode under applied voltage difference at the boundaries. The electric potential distribution can be further analyzed to simulate resistance changes with temperature. A three-dimensional multi-physics model consisting one liquid crystal elastomer film with uniaxial alignment and a sensing electrode was developed in COMSOL Multiphysics (Supplementary Fig. 14). The model can be divided into three parts: an LCON cube with a dimension of 3mm × 3mm × 0.1 mm, a sensing electrode with the dimension of 3 mm × 0.5mm × 0.03 mm, and an elliptical gap (a-semiaxis = 1 μm, b-semiaxis is 0.5 mm) penetrating the sensing electrode and the LCON film. During the construction of the model, we utilized experimental data and assumed that there is no gradient of order parameter in the thickness direction. For the simulation conducted, we selected an orthotropic linear elastic materials model for our aligned LCON. Depending on the type of resistive switch gate, the direction of the director is either parallel to the X-axis (STG) or Y-axis (SPG). Their corresponding material properties also change accordingly. In addition, an isotropic linear elastic materials model was used for the sensing electrode. The material parameters were either obtained from our experimental result or references[1,2]. To simulate the contact and separation process in the sensing electrode during deformation, a contact pair was added to the Solid Mechanics and the Penalty algorithm was used for computing the contact behavior. For the boundary conditions, in Solid Mechanics, the Rigid Motion Suppression and Prescribed Displacement (prescribed in z direction = 0 mm) were selected. In Electric Currents, a Ground boundary and Terminal boundary (0.001 V) were chosen. For the Stationary Solver, the direct solver MUMPS was applied for the solution at each iteration. The termination technique was selected as Tolerances, and its relative tolerance was set as 0.05.

Supplementary Figure

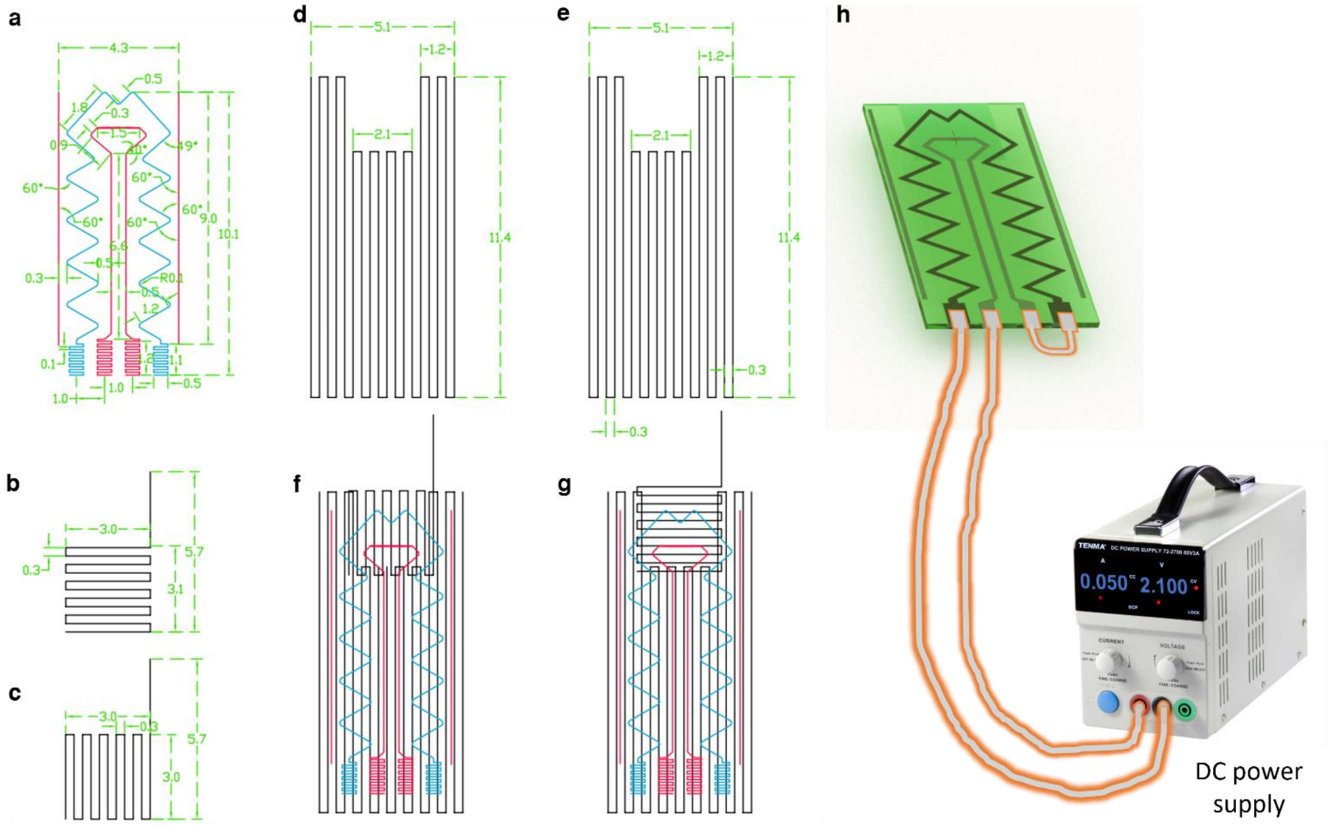

Supplementary Fig. 1, The specific dimension of the adaptive electronics unit and the usual connection of electrodes in the experiment. **a**, The dimension and printing paths for the sensing electrode (red), Auxiliary electrode (red), and actuating electrode (blue). **b, c**, The dimensions and print paths for printing the liquid crystal oligomer in the region of the logic switch, (**b**) for SPG, and (**c**) for STG. **d, e**, The dimensions and print paths for printing the liquid crystal oligomer in the rest region, (**d**) for SPG, and (**e**) for STG. **f, g**, The entire paths for printing the (**f**) SPG, and (**g**) STG. All dimensions are in millimeters. **h**, The 3D schematic of the adaptive electronics unit and the usual connection of the sensing and actuating electrode during the experiment.

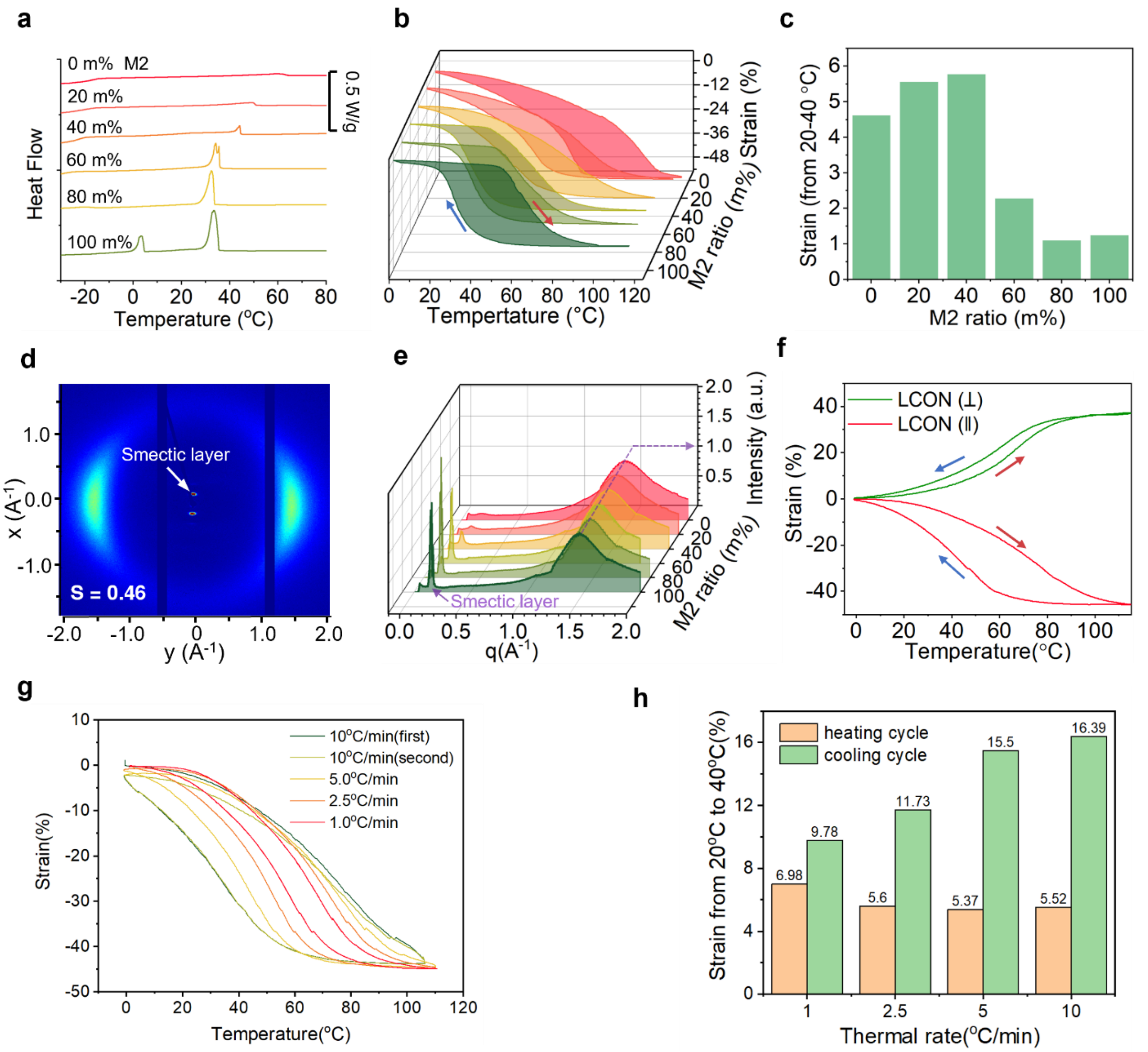

Supplementary Fig. 2 Optimizing the thermal strain of LCON by adjusting its phase transition temperature and phase morphology. **a**, The enthalpy curve of the liquid crystal oligomer with a different molar ratio of Molecule **2**. **b**, The thermal strain curves in the cured oligomer (parallel to the director) with a different molar ratio of Molecule **2**. The red and blue arrows indicate the heating and cooling process, respectively. **c**, The thermal strain in the desired range from 20 to 40 °C against the molar ratio of Molecule **2**. **d**, The 2D-WAXD pattern of the cured oligomer with 80 m% Molecule **2**. The annotated white arrow denotes the diffraction signal of the smectic layer in the LCON. **e**, Normalized azimuthal average intensity of the cured oligomer with different molar ratios of Molecule **2**. **f**, The anisotropic thermal strain of the cured oligomer film with 40 m% Molecule **2** was measured. Strain perpendicular to the director is indicated by the green line, while parallel strain is shown by the red line. The red and blue arrows indicate the heating and cooling process, respectively. The heating and cooling rate is 5 C°/min. The LCONs exhibit the notable hysteresis of thermal-mechanical response. This is because the transition kinetics of LC mesogens from a disordered to an ordered state is controlled by the cooling rate. The increased cooling rate will enlarge the hysteresis while the slowed cooling rate will reduce the hysteresis. This reason can be further proven by changing the thermal rate. **g**, The thermal strain curves in the cured oligomer (parallel to the director, 40 m% Molecule **2**) with a different thermal rate. **h**, The thermal strain in the desired range from 20 to 40 °C against the thermal rate. Higher thermal rates cause larger hysteresis in the strain.

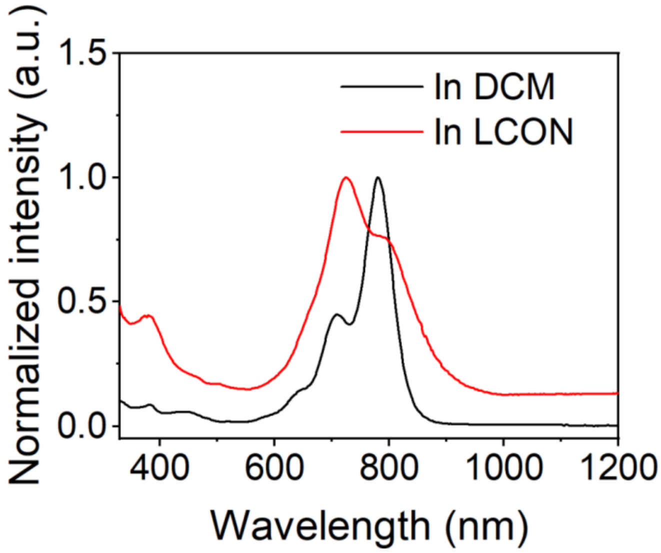

Supplementary Fig. 3, **The absorption of Molecule 3 in DCM and LCON.** Pure Molecule **3** exhibits a maximum absorption peak at 788 nm, which shifts to 700 nm when Molecule **3** is blended into LCON.

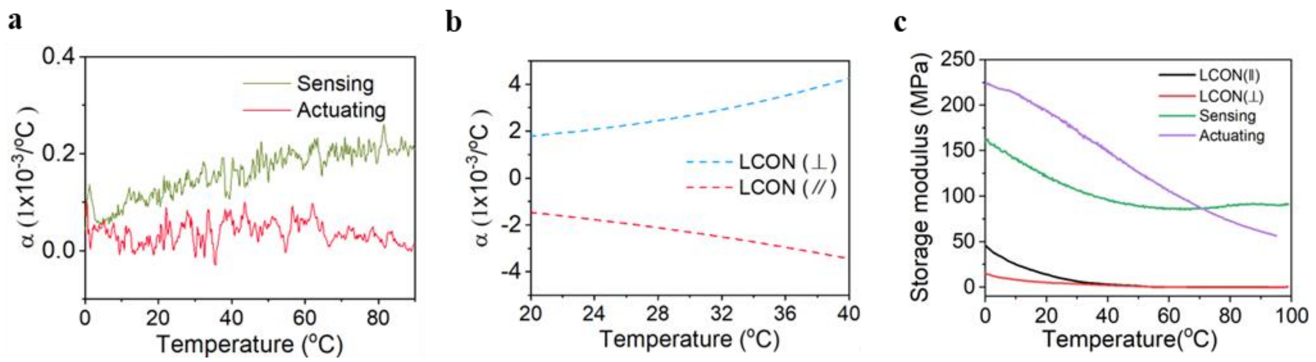

Supplementary Fig. 4, **The mechanical properties of the electrodes and LCON. a,** The thermal expansion coefficient of the sensing electrode (green) and actuating electrode (red) with varying temperature. **b**, The thermal expansion coefficient of LCON from 20-40 ^oC, where its absolute values are higher than 20 times that of electrodes in the temperature range from 20-40 ^oC. **c**, The storage modulus and loss modulus of the electrodes and LCON as a function of temperature.

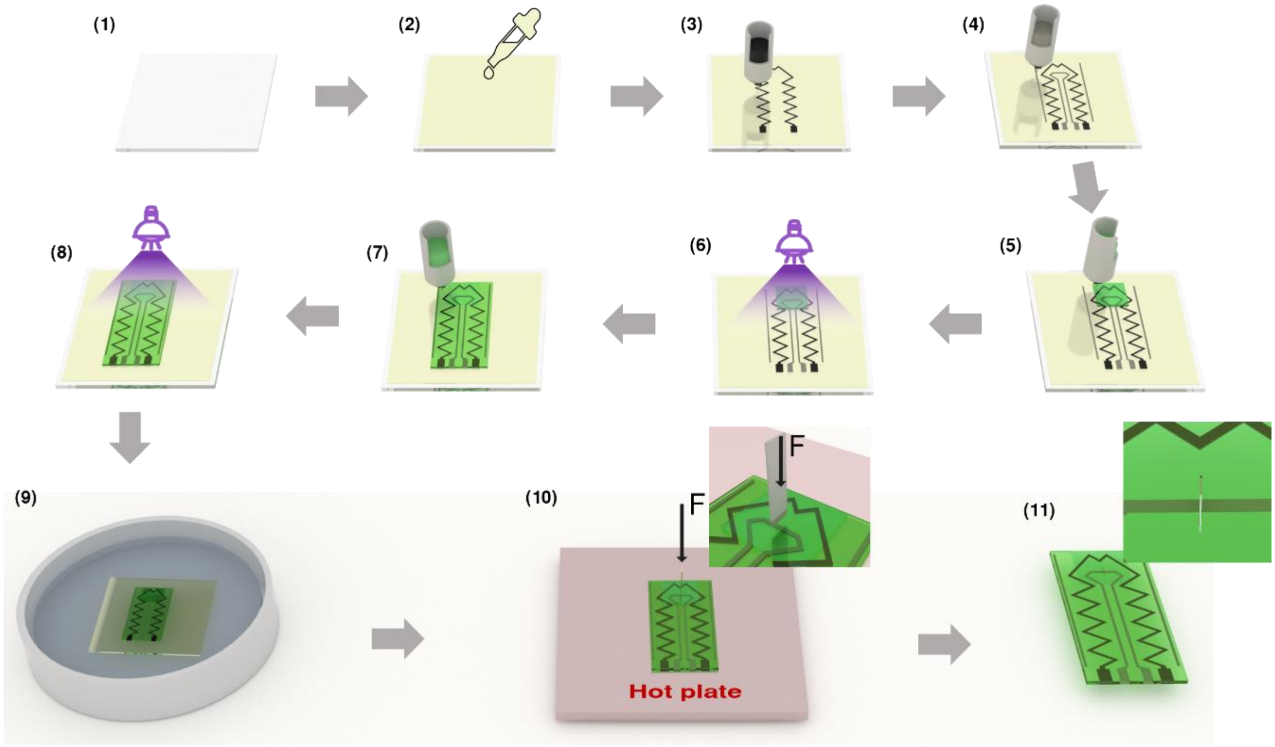

Supplementary Fig. 5, **The schematic fabrication process of the single adaptive electronic unit.** (1) Preparing substrate. (2) Spin-coating PVA. (3) Printing the actuation electrode. (4) Printing the sensing electrode and the auxiliary electrode. (5) Printing the oligomer in the region of a logic switch. (6) Photo-crosslinking the aligned oligomer above ambient temperature. (7) Printing the oligomer in the rest region. (8) Photo-crosslinking the oligomer at ambient temperature. (9) Dissolving PVA in water to obtain a free-standing film. (10) Using the mold to cut a micro-gap in the sensing electrode and LCON. (11) Obtaining a single adaptive electronic unit.

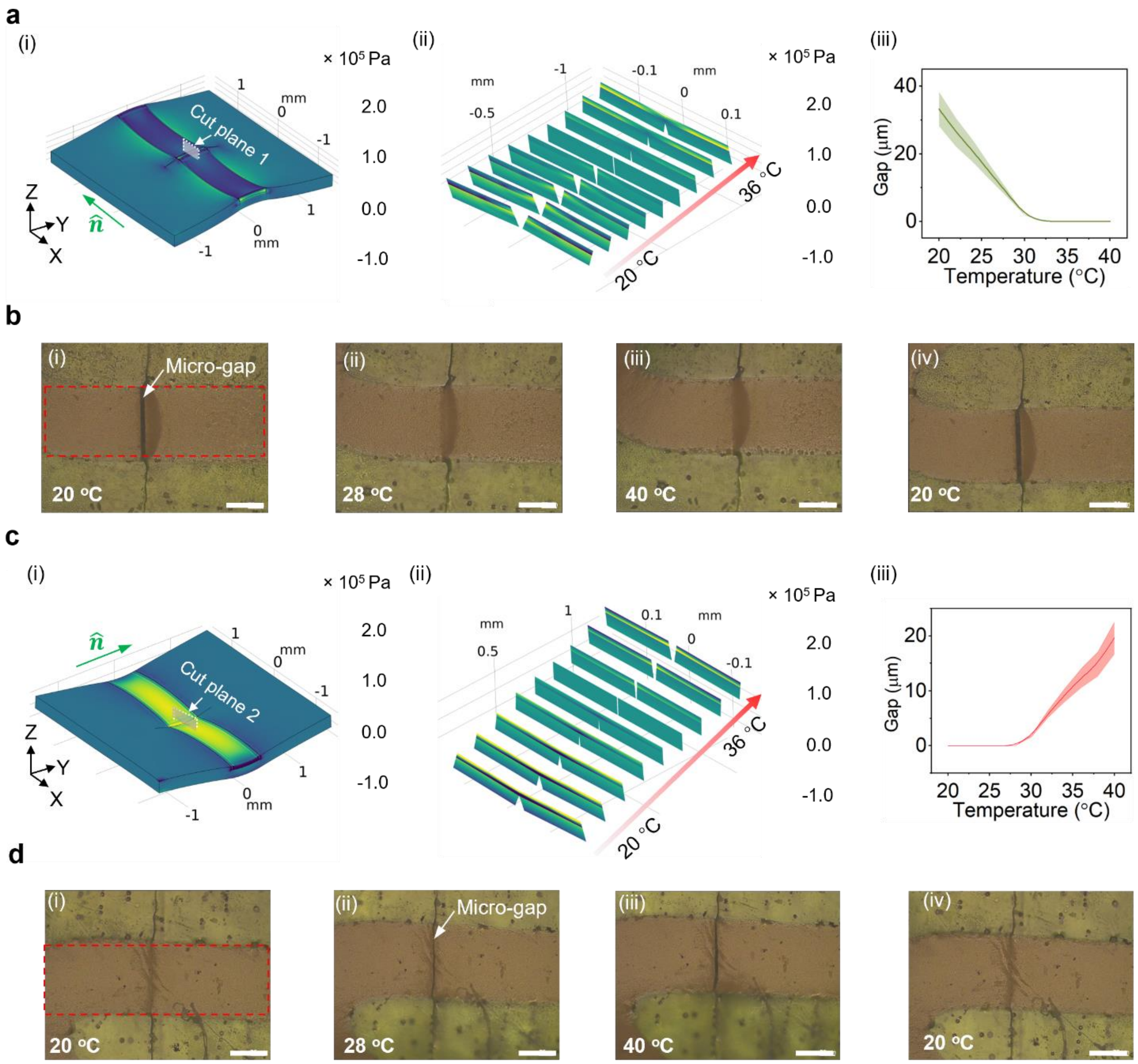

Supplementary Fig. 6 FEM and the experimental characterization of the reversible micro-gap in the adaptive unit. a, c, FEM results of the micro-gap change with temperature in (**a**) SPG and (**c**) STG. (i) The distribution of internal stress in the LCON device at room temperature. The green arrow indicates the molecular direction of LCON. (ii) The pressure distribution in the corresponding cut-plane in (i) changes with temperature. (iii) Gap size changes as a function of temperature. **b, d**, Experimental results of the micro-gap change with temperature in (**b**) SPG and (**d**) STG. The red-boxed areas represent where the sensing electrodes are located, the rest are LCON. The white arrows denote the position of the micro-gap. Scale bar, 150 μm

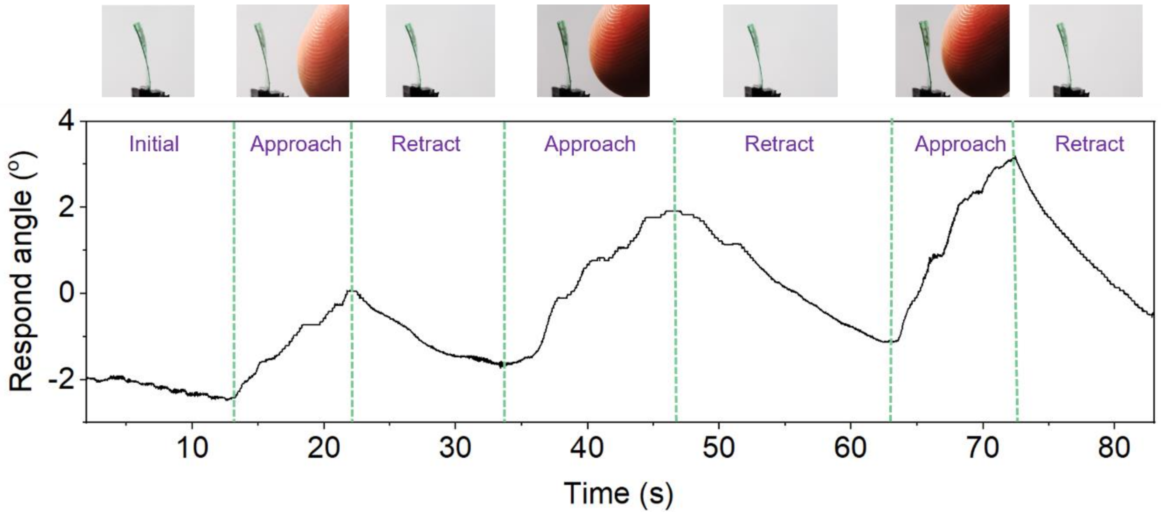

Supplementary Fig. 7, **The response of SPG when the actual temperature is below the threshold.** The SPG reverts to the initial state after the finger is retracted. Images of the experiment are shown at the top of the figure, while plots indicating the response angle in SPG are shown at the bottom. There are small hysteresis in the bending curve due to the limited cooling time. To eliminate this hysteresis, we can increase the cooling rate which can be controlled by using an active cool element, or prolong the cooling time.

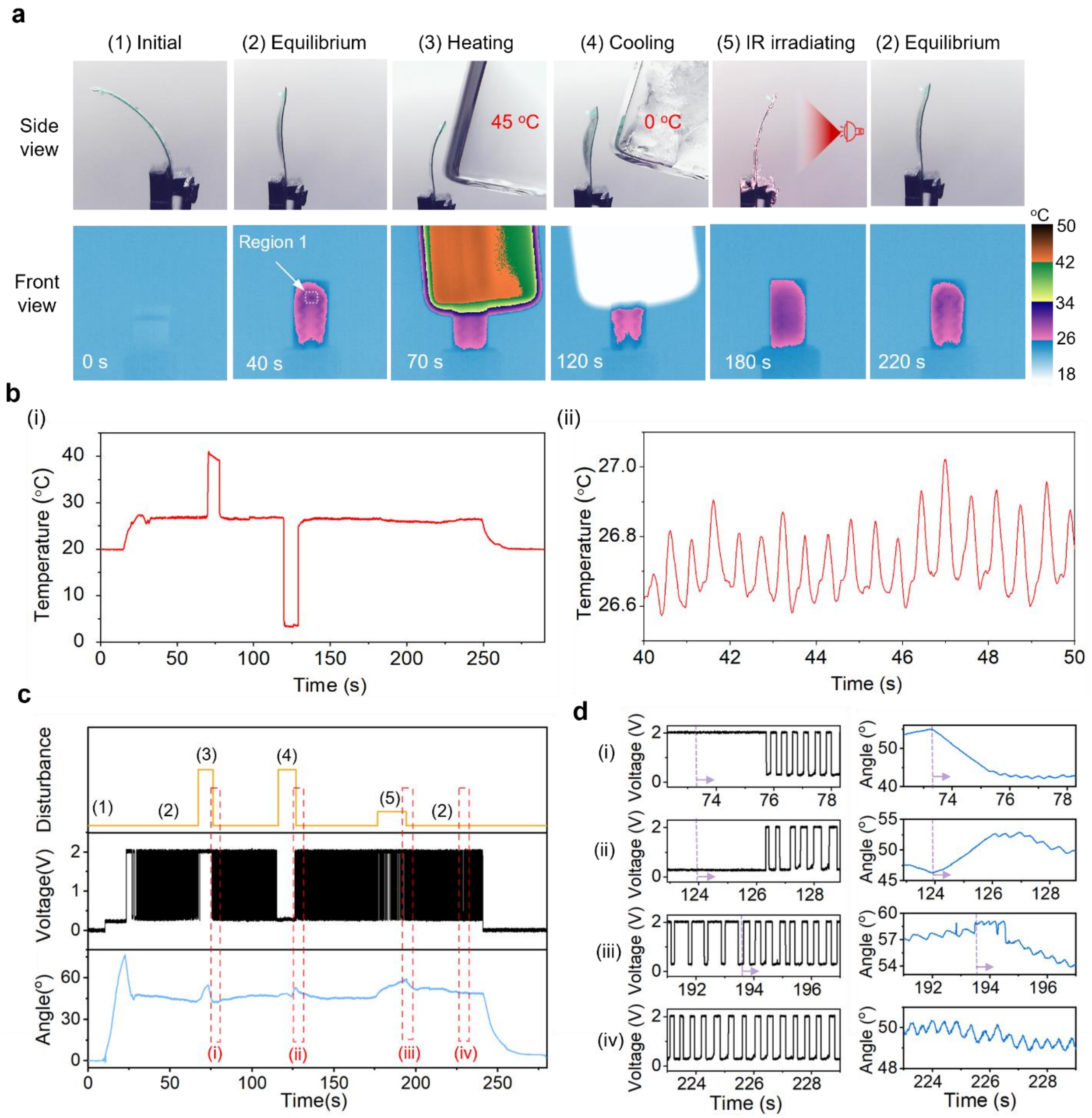

Supplementary Fig. 8 Characterization of the oscillation behavior in a single STG. a, Top, the snapshots of the oscillating behavior in STG. Botton, the corresponding snapshot of the thermal image during oscillation. **b**, The temperature changes over time. (i) Temperature changes in the "Region 1" of **a**, measuring from the front. (ii) The enlarged plot of (i) within 40-50 s. **c**, The response behavior of the STG when subjected to different disturbances. The positions of (1)-(5) correspond to STG at different stages in **a**. **d**, The enlarged plots correspond to regions (i) to (iv) in **c**. The purple arrows indicate that the disturbances are starting to leave the negative feedback loop.

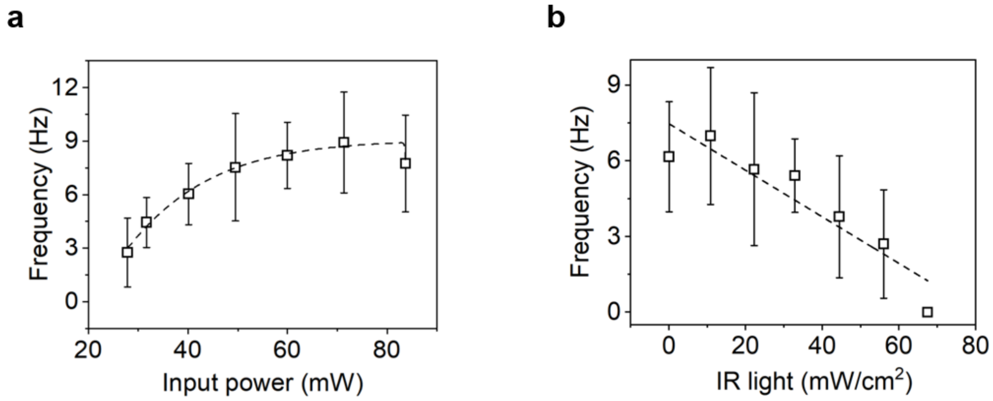

Supplementary Fig. 9, **Adjusting the oscillation behavior in the STG. a,** The oscillation frequency increases with the increase of input power. The error bar indicates the standard deviation of the oscillatory frequency over 30s. **b**, The oscillation frequency decreases with the increase of IR light intensity. The input power is 40.12 mW. The error bar indicates the standard deviation of the oscillatory frequency over 30s.

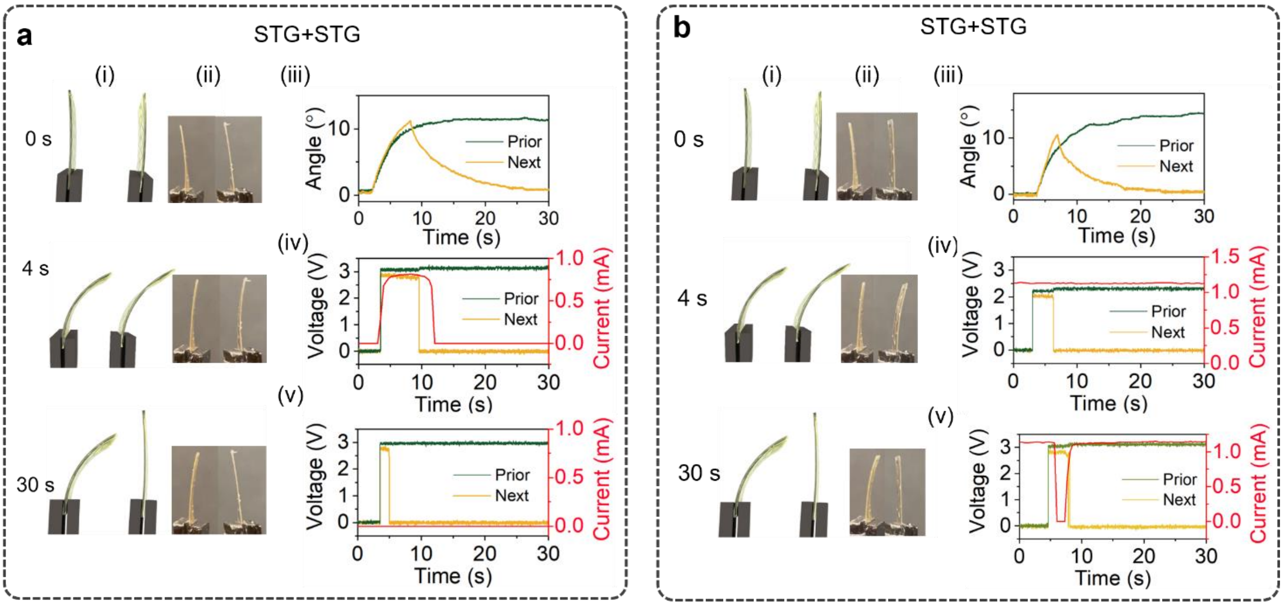

Supplementary Fig. 10 Signal cascade transmission between multiple adaptive electronic units. a, Signal cascade transmission between STG and SPG. (i) The schematics of their deformation. (ii, iii) The corresponding experimental results. (iv) The corresponding voltage and current changes during signal transmission when the termination rate of the prior unit is lower than the transmission rate of the next unit, (v) The voltage and current changes during signal transmission when the termination rate of the prior unit is higher than the transmission rate of next unit. **b**, Signal cascade transmission between two STGs. (i) The schematics of their deformation. (ii, iii) The corresponding experimental results. (iv) The corresponding voltage and current changes during signal transmission when the termination rate of the prior unit is higher than the transmission rate of the next unit. (v) The voltage and current changes during signal transmission when the termination rate of the prior unit is lower than the transmission rate of the next unit.

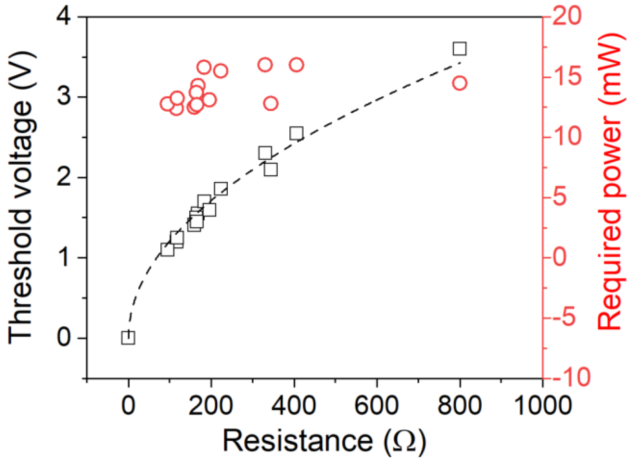

Supplementary Fig. 11, The threshold voltage change and required power input against the resistance of the actuating electrode.

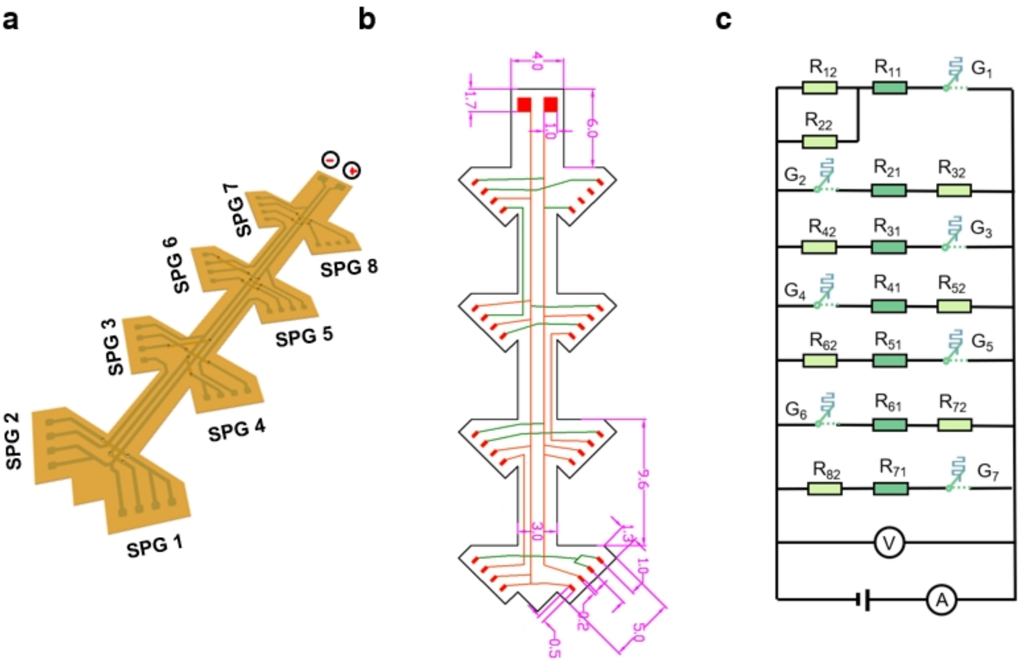

Supplementary Fig. 12, **The details of designing a flexible PCB board for the artificial Mimosa. a**, The 3d model of the PCB. **b,** The 2D dimension of the PCB. All dimensions are in millimeters. **c**, The corresponding circuit of the artificial Mimosa. The $\mathbf{R_{xy}}$ represents the resistor in the adaptive unit, where the subscript “x” corresponds to the order in the artificial Mimosa leaf, and the subscript “y” corresponds to the resistor in the sensing electrode (marked “1”) and actuating electrode (marked “2”) respectively. $\mathbf{G_{x}}$ represents the logic switch of the SPG in different artificial leaflets.

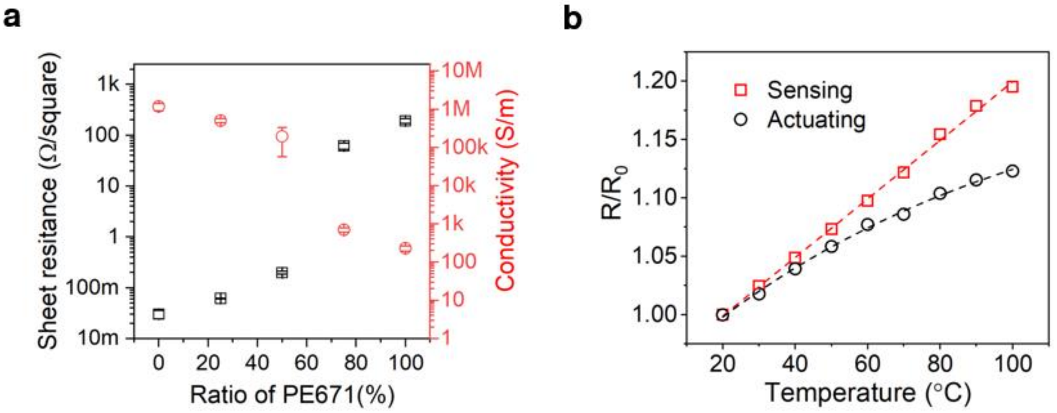

Supplementary Fig. 13, Characterization of the electric properties of the cured conductive ink. **a**, The sheet resistance and conductivity of the blending conductive ink change with the ratio PE671. **b**, The resistance of the sensing electrode and actuating electrode change with temperature after printing and curing them onto the glass substrate. R_0 indicates the resistance at room temperature.

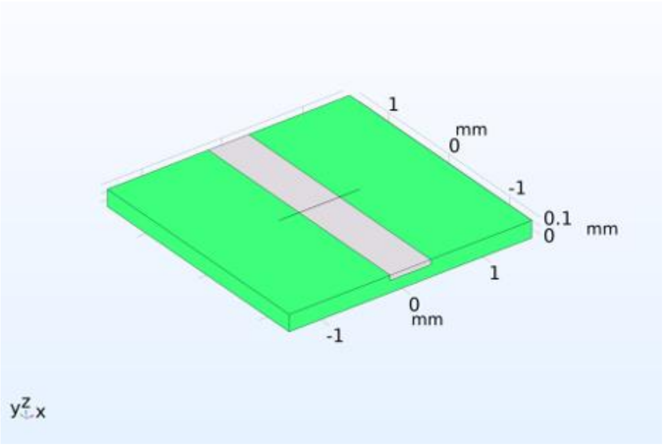

Supplementary Fig. 14, The 3D model used for simulation of the reversible micro-gap in COMSOL Multiphysics. The grey part indicates the electrode and the green part indicates the LCON

Supplementary References

1. Oh, S. W. *et al.* Measuring the five elastic constants of a nematic liquid crystal elastomer. *Liq. Cryst.* **48**, 1–11 (2020).

2. Cang, Y. *et al.* On the origin of elasticity and heat conduction anisotropy of liquid crystal elastomers at gigahertz frequencies. *Nat. Commun.* **13**, 1–12 (2022).
