# Supplementary material for: Advancing interactive systems with liquid crystal network-based adaptive electronics
Source: Nat Commun. 2024 May 17;15:4191. doi: 10.1038/s41467-024-48353-7 (PMC11101476; doi:10.1038/s41467-024-48353-7)
Supplement: Supplementary file 3 — Description of Additional Supplementary Files [file 41467_2024_48353_MOESM3_ESM.docx]

File Name: Supplementary Video 1
Description: The sequential folding behavior in *Mimosa pudica Linn*

File Name: Supplementary Video 2
Description: Reversible micro-gap in SPG

File Name: Supplementary Video 3
Description: Reversible micro-gap in STG

File Name: Supplementary Video 4
Description: The adaptive bending behavior in SPG

File Name: Supplementary Video 5
Description: The adaptive oscillation behavior in STG

File Name: Supplementary Video 6
Description: Artificial *Mimosa* containing eight SPGs

File Name: Supplementary Video 7
Description: Collective oscillation behavior in multiple adaptive units
